# A Traffic Splitting Algorithm for Load Balancing in Tor

**DOI:** 10.3390/e24060807

**Published:** 2022-06-09

**Authors:** Xiance Meng, Mangui Liang

**Affiliations:** 1 Institute of Information Science, Beijing Jiaotong University, Beijing 100044, China; mengxiance@bjtu.edu.cn; 2 Beijing Key Laboratory of Advanced Information Science and Network Technology, Beijing Jiaotong University, Beijing 100044, China

**Keywords:** anonymous network, Tor, anonymity, load balancing

## Abstract

As the most popular anonymous communication system, Tor provides anonymous protection for users by sending their messages through a series of relays. Due to the use of the bandwidth-weighted path selection algorithm, many more users choose routers with high bandwidth as relays. This will cause the utilization of high bandwidth routers to be much higher than that of low bandwidth routers, which will bring congestion risk. The Quality of Service (QoS) is difficult to guarantee for users who need delay-sensitive services such as web browsing and instant messaging. To reduce the average load of routers and improve the network throughput, we propose a circuit construction method with multiple parallel middle relays and conduct a dynamic load allocation method. The experiment demonstrates that our proposed method can provide better load balancing. Compared with other multipath anonymous communication networks, our proposed method can provide better anonymity.

## 1. Introduction

With the rapid development of Internet technology, more and more Internet users pay attention to user privacy. Various anonymous communication systems have been proposed in recent years, such as the Anonymizer [1], DC-Net [2], Crowds [3], Tarzan [4], LAP [5], HORNET [6], TARANET [7], and Tor [8]. The Second-Generation Onion Router (Tor) is the most widely used anonymous communication system with low latency, which is a distributed overlay network developed based on the existing general Internet. It encrypts the transmitted data in layers through a series of relays and transmits data to the receiver. Each node in the anonymous path only knows its predecessor node and successor node but does not know the information of other nodes in the path, thus protecting the anonymity of the connection. However, for delay-sensitive applications, such as web browsing and instant messaging, the delay of Tor leads to poor user experience [9]. Furthermore, this may cause some users to exit, which will reduce the size of the anonymity set, thus affecting the anonymity for all users [10,11].

Recent studies have found that the following factors mainly cause Tor’s delay. First, users using video streaming applications, P2P applications, and other applications occupy a lot of bandwidth resources [12]. Second, Tor has a longer path and requires multiple encryptions and decryptions compared with the typical network. The last is that the performance of nodes in Tor is unreliable [13].

We find that the layered encryption and decryption scheme can guarantee Tor’s security based on the above aspects. Furthermore, Tor is the most popular system for providing anonymity on the Internet. It is normal for many users to use high-bandwidth applications. In order to improve the performance of Tor, Mohsen et al. [14] proposed a path selection algorithm considering the geographical location information and bandwidth of nodes to reduce the delay. Armon et al. [15] proposed PredicTor, which can dynamically avoid selecting congested nodes and long-distance paths. However, these methods will bring a small amount of anonymity loss. Recently, Tor has adopted a new method to deal with congestion [16,17]. This method will track the RTT measurement value of each circuit and compare it with the threshold to determine whether it is congested and update the path. This method can improve Tor’s load balancing and congestion problems. However, this still cannot change that low-bandwidth nodes have poor load capacity. Therefore, we consider using a multipath method to solve this problem. In Tor, multipath routing has the following advantages:

- Improve load balancing [18]. Using multipath simultaneously can reduce the load assigned to each OR.
- Increase throughput [19]. The throughput of multipath users can achieve up to the sum throughput of all circuits, which is greater than the throughput of a single path.

We present TSMMR, a traffic splitting mechanism with multiple middle relays in parallel. The traffic sent by the sender splits into several streams by entry relay and will forward in parallel through multiple middle relays. Finally, the traffic will combine in the exit relay. We only have one entry relay and one exit relay at the two ends. The minimum bandwidth of each node on each path is the path’s capacity, and the load of each path is allocated according to its capacity. We also propose a new performance evaluation metric, and, according to the evaluation, our method can reduce network utilization compared with Tor and thus reduce the load on nodes. Conflux [20], mTor [21], and TrafficSliver [22] have used multipath in Tor in recent years. Among them, Conflux and mTor can improve the performance in Tor. TrafficSliver involves a multipath method to resist website fingerprinting attacks, but it sacrificed some bandwidth and latency overheads. Therefore, we only compare the performance and anonymity with those of Conflux and mTor in this paper.

This work’s significant contributions may be summarized as follows:

(1) Better balance of the utilization of various nodes to effectively improve the congestion problem of high-bandwidth nodes, as well as the problem of bandwidth scarcity and reducing the load of high-bandwidth nodes.
(2) A traffic splitting algorithm is proposed.
(3) A new performance evaluation metric is proposed for performance analysis.
(4) Anonymity can be guaranteed.

The rest of this paper is organized as follows. In Section 2, we present the related work. Then, in Section 3, we present our proposed approach. Next, we show our performance evaluation in Section 4. We analyze the anonymity in Section 5. Finally, we conclude our work in Section 6.

## 2. Related Work

### 2.1. Tor

**Figure 1:**
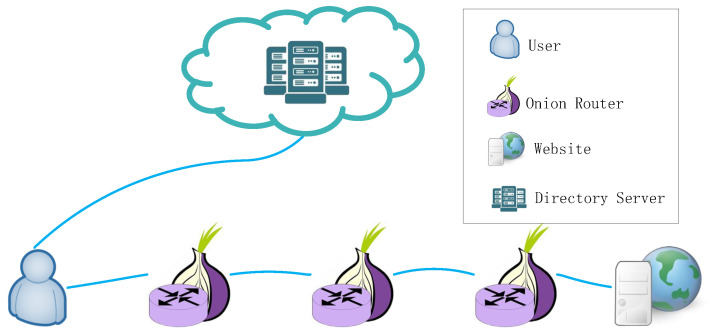
Tor architecture.

As the most popular anonymous communication network with low latency, Tor usually chooses three nodes as relays to construct a path from the client to the destination, commonly called a circuit. These three kinds of nodes are Onion Proxies (OPs), Onion Routers (ORs), and Directory Servers (DSs). As shown in Figure 1, OPs run on a user’s machine to fetch directories and construct circuits across the network. ORs of the circuit are responsible for relaying traffic to destinations or other relays. As a group of trusted and reliable servers, Directory Servers are deployed in the Tor network as centralized, and are responsible for collecting each OR’s IP address, public key, policies, and bandwidth value of the OR. Generally, as the first router (entry guard), they can protect Tor from traffic analysis attacks, including predecessor attacks, statistical analysis attacks, and passive AS level association attacks. In order to enhance these resistances, Tor increases the cycle of rotating entry guards to 9 months and changes from using three entry guards to a single, fast entry guard [23]. The original definition of fast entry guard is higher than the median bandwidth or 250 kB/s. Now, the threshold is increased to 2 MB/s. The strategy for Tor to select other nodes, such as middle relay and exit nodes, is through the bandwidth-weighted path selection algorithm. For example, the bandwidth of node *i* is $B_{i}$. Then, the probability of the node being selected is:

(1) $$Q_{i}=\frac{B_{i}}{\sum_{i=1}^{n}B_{i}}.$$

The number of nodes is *n*. The strategy for Tor to select other nodes is through the bandwidth-weighted path selection algorithm.

### 2.2. Conflux

Conflux [20] and mTor [21] are extensions to vanilla Tor that utilize multiple paths to improve the user experience. In Conflux, the OP creates two or more circuits with the same exit OR. The client will split traffic and transmit it across multiple circuits, dynamically measuring the throughput and latency of each circuit. If the throughput or the latency on a path is high, the client will reduce the load on that path. Since the split traffic will arrive out of order, the client adds a 4-byte sequence number to each cell. Finally, the traffic is reordered and combined by the exit OR. This method requires incremental deployment to upgrade the client and exit OR. It improves performance for low-bandwidth clients such as bridged users. However, since Conflux uses more nodes than Tor, it will have a higher path compromise rate.

### 2.3. mTor

In addition, mTor also involves a multipath anonymous communication network similar to Conflux. However, the number of circuit configurations is *m* as the parameter. In mTor, it selects a group of new low-bandwidth routers as relays, constructs multiple circuits to form anonymous tunnels for bulk data transmission, and uses an active congestion detection mechanism to prevent slow circuits from becoming the bottleneck of the whole tunnel to improve the performance of bulk data transmission.

## 3. Traffic Splitting Mechanism with Multiple Middle Relays in Parallel

Low-resource routing attack is an irresistible attack for Tor [24]. When both the entry and exit nodes on the anonymous path are malicious nodes, an adversary can collude with them to compromise Tor’s anonymity. We find the nodes with high bandwidth are much more utilized than nodes with low bandwidth as most users tend to choose stable and fast nodes as Tor routers [25]. This causes two problems. The first is that more users select high-bandwidth nodes, so their bandwidth utilization and load will be high, resulting in risks such as congestion and increased delays. Second, nodes deployed or claimed by the adversary to have high bandwidth will be more likely to be selected, making the probability of both entry and exit nodes being malicious nodes higher. Therefore, we consider improving the utilization of low-bandwidth nodes to solve the above two problems. However, due to the poor capacity of low-bandwidth nodes, the multipath method should be considered to split the load. Next, we will describe the details of our proposed TSMMR.

### 3.1. Circuit Construction

**Figure 2:**
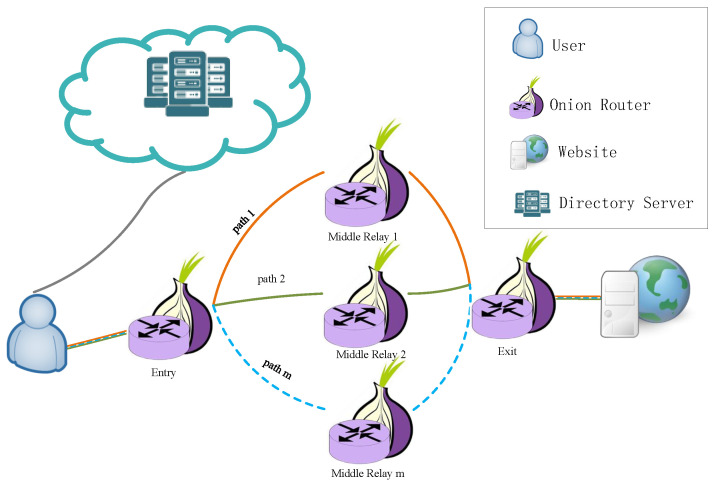
TSMMR architecture. The user splits the traffic and transmits it through *m* different paths to the website.

In Figure 2, our proposed anonymous communication network has *m* paths with different middle relays. Common circuit construction strategies include random selection of nodes [26], geographical selection of nodes [27], and the bandwidth-weighted path selection algorithm [28]. The random selection of nodes strategy has high anonymity but cannot ensure bandwidth, so it is not easy to provide QoS to users. The geographical selection of nodes strategy makes it easy to expose the geographical location of users, thus reducing the anonymity of the system. In TSMMR, traffic is transmitted through multiple paths. Where the entry node and exit node are aggregation nodes for the traffic, we prefer to select high-bandwidth entry nodes and exit nodes to construct circuits. We propose a hybrid node selection strategy to meet the needs of different relay nodes. Next, we will first present our proposed node selection strategy.

**Selection of entry guard.** The entry node is the first hop on the anonymous communication path, and this node knows the identity information of the sender. If an adversary controls the entry node, it will directly reveal the sender’s identity. Research shows that rotating the entry node too frequently will make it easier to select the node controlled by the adversary as the entry node [29]. Therefore, nodes are generally rotated infrequently to defend against end-to-end correlation attacks and can be retained for 60 days to 9 months. Thus, the uptime of the entry node should be higher than that of most routers. In addition, the node will have a high probability of being used multiple times during long-term running. Therefore, we choose nodes above the median bandwidth (currently about 2MB/s). The user first downloads the information of the alternative nodes from the DS and excludes from it the nodes that do not satisfy the above conditions. Subsequently, the node is selected as the entry node using the bandwidth-weighted path selection algorithm. The specific description of the bandwidth-weighted path selection algorithm can be seen in Algorithm 1. Suppose there are *n* ORs and the bandwidth of a node is $B_{i}$. According to Formula (1), the probability of this node being selected as an entry node is $Q_{i}$.

**Selection of exit node.** The exit node is the node on the anonymous communication path that knows the receiver’s identity information and is closely related to the receiver’s anonymity guarantee. In addition, this node is also the aggregation node in TSMMR. In order not to decrease the throughput on the communication path, the bandwidth of the exit node should be higher than the median bandwidth. After excluding the nodes that do not meet the requirements, the user selects the exit node using the bandwidth-weighted path selection algorithm.

**Selection of middle nodes.** The entry and exit nodes of Tor are quite important. However, the role of middle relays on the anonymous communication path is also crucial. When a middle relay fails due to insufficient bandwidth, the sender will reselect a middle relay to construct a new communication path. However, it will increase the communication cost and increase the communication delay. Therefore, we consider choosing multiple middle relays, where each middle relay and the entry and exit nodes can construct a circuit and multiple circuits multiplex the entry and exit nodes. The multiple circuit construction method improves the network throughput on both sides of the communication and allows for fast traffic redistribution in case of failure of the middle relay node. The bandwidth of this node need not be higher than the median bandwidth. Using a bandwidth-weighted path selection algorithm in Algorithm 1, the user can select the middle relay directly among the running nodes.
**Algorithm 1** Bandwidth-Weighted Path Selection Algorithm.**Require:** A list of nodes node_list fetched from Directory Servers
**Ensure:** A chosen router
1: **for**
i←0 to node_list.size
**do**
2:     $B_{i}\leftarrow node\_list\left[i\right].bw$
3:     $bw\_list\leftarrow bw\_list\cup B_{i}$
4:     $BW\leftarrow BW+B_{i}$
5: **end for**
6: Rnd←rand()%BW
7: **while**
T<Rnd
**do**
8:     $T\leftarrow T+bw\_list_{i}$
9:     i←i+1
10: **end while**
11: **return**
router←node_list[i]


### 3.2. Traffic Splitting

Since we construct circuits using multiple middle relays with different bandwidths, the average traffic splitting may lead to bandwidth redundancy in high-bandwidth relays and congestion in low-bandwidth relays. We propose an adaptive traffic splitting method in a round-robin fashion to solve this problem. Assume that the size of each data stream sent by the sender is *D*. When the sender first initiates a communication request, it asks for the used and advertised bandwidths of the middle relays in turn. As a result, the used bandwidths of the middle relays are $\left\{Bu_{1},Bu_{2},\ldots ,Bu_{m}\right\}$. The advertised bandwidths of the middle relays are $\left\{B_{1},B_{2},\ldots ,B_{m}\right\}$, where *m* is the number of paths, and *B* is the sum of the bandwidths of all middle relays. We can see the details in Algorithm 2.

(2) $$B=\sum_{i=1}^{m}B_{i}.$$

For each circuit $C_{i}\left(i\in \left[1,m\right]\right)$, the allocated traffic is:

(3) $$D_{i}=D*\frac{B_{i}}{B}.$$

As the number of users grows, the probability of a circuit being multiplexed increases. Thus, it increases the risk of node congestion. We assume that the used bandwidth of a middle relay $R_{t}$ is $Bu_{t}$, the advertised bandwidth is $B_{t}$, and the traffic allocated to this node is $D_{t}$. If there exists

(4) $$Bu_{t}+D_{t}>B_{t},$$

we consider that the middle relay $R_{t}$ is no longer suitable for constructing circuits for an anonymous communication network. Therefore, the traffic needs to be reallocated, where the node $R_{t}$ will be allocated traffic of $D_{t}=0$. For other circuits $C_{i}\left(i\in \left[1,m\right],i\neq t\right)$, the traffic is still allocated in proportion to the bandwidth. The number of paths is m−1 until the user finds a new middle relay. Our way of coping with node failures enables timely data transmission using other paths in case of node failure or congestion. Compared with a single path, it saves the time spent re-finding nodes to construct circuits. Our traffic splitting approach can provide better load balancing by allocating corresponding loads to nodes with different bandwidths in the network.

Due to the multipath routing scheme, the traffic will split into different streams along different paths. The sender adds the sequence number to each stream, and the receiver can reorder the traffic according to the sequence number to obtain the complete data. In addition, the exit OR is responsible for buffering the out-of-order data.
**Algorithm 2** Traffic Splitting Algorithm.**Require:** A list of middle relays middle_list and the traffic LD sent by the sender
**Ensure:** Traffic ld_list which is allocated at each relay
1: **for**
i←0 to *m*
**do**
2:     $B_{i}\leftarrow middle\_list\left[i\right].bw$
3:     $B\leftarrow B+B_{i}$
4: **end for**
5: **for**
i←0 to *m*
**do**
6:     $ld\_list\left[i\right]\leftarrow LD*B_{i}/B$
7: **end for**
8: **return**
ld_list


## 4. Performance Evaluation

We conduct a simulation to evaluate the performance improvements of our proposed method. The results show that our method can improve reducing the network utilization of most nodes, and it will provide benefits for load balancing.

### 4.1. Performance Metrics

In general, high-bandwidth routers are capable of carrying more traffic. As a router carries high traffic, its capacity to process data will decrease, which increases latency and thus increases the risk of congestion on the path. Therefore, we use the network utilization of nodes as a metric to evaluate network scalability. We conducted a simulation and compared it with other anonymous networks. The results show that our proposed TSMMR can carry more traffic load and has better scalability.

### 4.2. Simulation Design

Our simulation is implemented on OMNeT++ [30]. We design three types of nodes: user, website, and Onion Router. The Onion Router contains the entry node, the middle relay, and the exit node. The Onion Proxy runs on the user node and is responsible for selecting nodes and constructing circuits in the anonymous communication network. The user sends traffic to the website through the OR. We simulate Conflux, mTor, Tor, and TSMMR using different circuit construction methods.

To make the simulation environment close to the actual network environment, we download Tor’s consensus network status document as the original data for our simulation [31]. This document records the bandwidth and online time of the nodes in Tor.

We use different path selection algorithms to select nodes from these nodes for constructing circuits. Different types of applications have different bandwidth requirements. To make the experiments more convincing, we set different sending rates for the user nodes to simulate different kinds of user requirements. For example, the lower sending rate can simulate applications such as real-time streaming. The higher sending rate can simulate applications such as video browsing and large file downloads. In Tor, when communication initiates, we use the send rate as the load for each Onion Router since there is only one communication path. In other multipath anonymous communication networks, the load on each node is calculated based on different traffic splitting algorithms. Then, based on the load and bandwidth of each node, we can obtain the bandwidth utilization of each node, which is the ratio of the total load to the bandwidth on that node.

As the scale of users increases, there will be many users running simultaneously, so there will be some ORs being multiplexed. When the bandwidth utilization of an OR is higher, it means that the performance of this node is more severely affected. Under the same conditions, we believe that a network has better scalability when the percentage of nodes with higher bandwidth utilization is low. We simulate the bandwidth utilization of the whole network when 10,000 users use different anonymous networks separately.

### 4.3. Results

Because the anonymous network has a congestion control mechanism, in the actual simulation process, we found that only a few nodes with very low bandwidth are prone to the situation that the bandwidth occupation cannot meet the demand. Therefore, we compared the cumulative fraction for different bandwidth utilization. Where bandwidth utilization is the ratio of an OR’s used bandwidth to its total bandwidth, the cumulative fraction is the number of ORs less than the specified bandwidth utilization as a percentage of the total number of ORs. Generally speaking, anonymous networks with a higher cumulative fraction with lower bandwidth utilizations have higher scalability.

**Figure 3:**
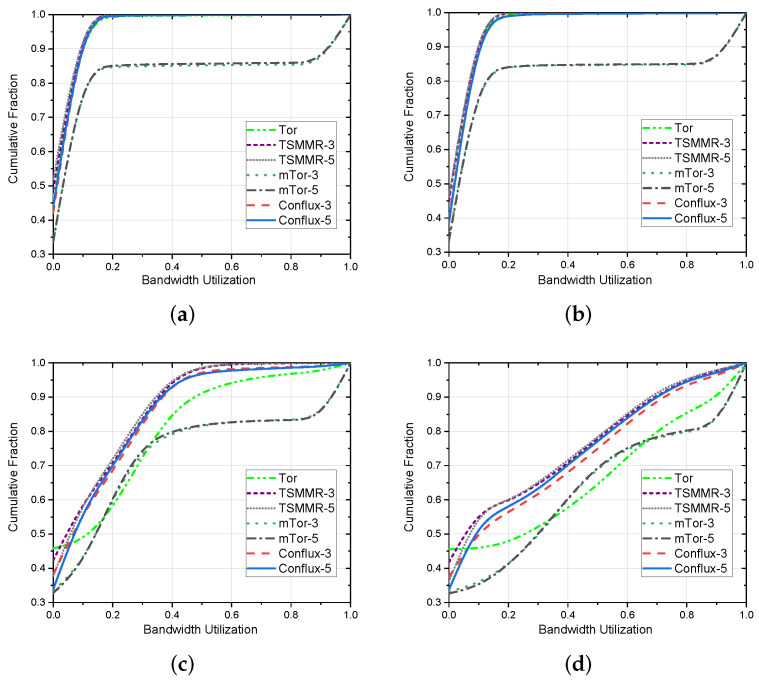
Cumulative fraction of node bandwidth utilization for different anonymous communication networks with different bandwidth requirements. (**a**) 50 kB/s sending rate. (**b**) 100 kB/s sending rate. (**c**) 500 kB/s sending rate. (**d**) 1000 kB/s sending rate.

As shown in Figure 3, we compare Tor with mTor, Conflux, and TSMMR. Our method has a higher cumulative fraction at low bandwidth utilization, which means fewer nodes with high bandwidth utilization in TSMMR. It also demonstrated that our method has better scalability and can accommodate more users with better congestion control.

We define nodes with bandwidth utilization below 30% as light-load nodes and nodes with bandwidth utilization above 80% as high-load nodes. High-load nodes are more likely to cause link congestion due to high latency. In Table 1, Table 2, Table 3 and Table 4, we can see that the percentage of light-load nodes is higher in TSMMR than in other anonymous communication networks. Moreover, the percentage of high-load nodes in TSMMR is lower than that of other anonymous communication networks. Therefore, we can prove that TSMMR has better load balancing capability and can provide better congestion control for users.

## 5. Anonymity Analysis

The anonymous communication networks mentioned in this paper are all based on Tor. When the adversary controls both ends of the circuit, the anonymity of the circuit may be compromised by time analysis. The adversary we have assumed depends upon the threat model proposed by Syverson et al. [32]. The entry node knows the client’s IP address in anonymous communication networks, while the exit node knows the server’s IP address. When the adversary controls both nodes, the adversary can use traffic analysis to confirm the communication relationship of the communication, thereby destroying the anonymity of the link [24,33]. In this section, we first introduce the threat model and compare the potential for path compromise under a given adversary with other anonymous communication networks. Finally, we compare the anonymity degree of these anonymous communication networks.

### 5.1. Threat Model

**Figure 4:**
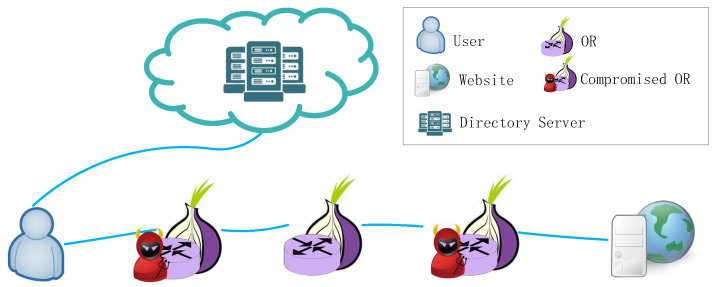
Threat model: The adversary controls the entry node and exit node on the path.

Tor is deployed on a real network and is the most popular anonymous communication network in the world. As a result, it is difficult to avoid some malicious adversaries to evaluate the anonymity of anonymous communication systems and resist some adversaries. First, we need to define the capabilities of the adversary. We assume that some of the Tor routers are controlled by the adversary. As shown in Figure 4, the OR compromised by the adversary can observe the network traffic, so the adversary can apply timing analysis [34,35] to destroy the network’s anonymity. However, traffic will not be modified, deleted, or delayed. In addition, the percentage of routers or bandwidth controlled by these malicious adversaries cannot exceed 20% [20].

### 5.2. Path Compromise

In Tor, every relay can only obtain the node information before and after. Therefore, only the entry node can identify the sender, and only the exit node can identify the receiver. When both the entry node and the exit node are under the adversary’s control, the anonymous communication network is considered compromised. We define P(Compromised) as the probability that an entry node and an exit node on an anonymous communication path are controlled by an adversary simultaneously.

In Tor, we can calculate P(Compromised) as follows:

(5) $$P\left(Compromised\right)=f_{xbw}\cdot f_{gbw}.$$

Here, $f_{gbw}$ is the proportion of the bandwidth of the entry nodes controlled by the adversary to the total bandwidth of Tor, $f_{xbw}$ is the proportion of the bandwidth of the exit nodes controlled by the adversary to the total bandwidth of Tor. As Conflux and mTor have multiple entry nodes, the compromised probability is different from Tor.

In Conflux and mTor, we can calculate P(Compromised) as follows:

(6) $$P\left(Compromised\right)=f_{xbw}\cdot \left(1-{\left(1-f_{gbw}\right)}^{m}\right).$$

Here, *m* is the number of entry nodes used in the multipath, $f_{xbw}$ and $f_{gbw}$ have the same definition as above. Like Tor, TSMMR has only one entry node and one exit node. As a result, as Formula (5) shows, the P(compromised) of TSMMR is the same as Tor. In addition, mTor and Conflux have a similar P(compromised).

**Figure 5:**
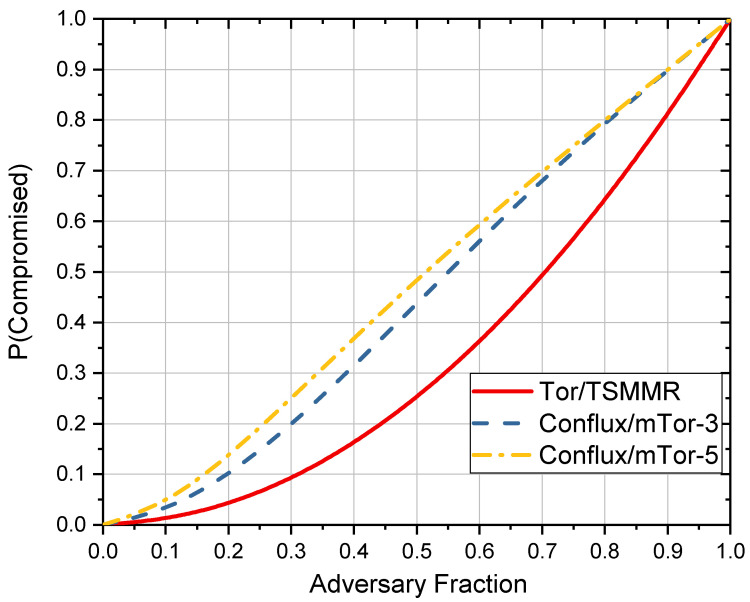
Compromise probability for different anonymous communication networks.

The comparison of P(compromised) between the several anonymous communication networks mentioned here is shown in Figure 5. The probability of being compromised increases with the adversary controlling more nodes. Whether the multipath number is 3 or 5, both Conflux and mTor have higher P(Compromised) than Tor and TSMMR.

### 5.3. Anonymity Degree

As mentioned above, we analyze the anonymity of anonymous networks primarily for adversaries who can control some of the network nodes. To be able to adopt a more general manner of describing anonymity in anonymous networks, we define the anonymity degree based on entropy [36].

**In Tor and TSMMR:** We assume that there are *N* nodes in the anonymity network. Then, the maximum value of the anonymity set is *N* for the adversary. Suppose the adversary cannot exclude any node in the anonymity set. Then, the maximum entropy of *N* users is:

(7) $$H_{M}=-\sum_{i=1}^{N}p_{i}\cdot \log_{2}p_{i}.$$

Here, *M* is the maximum value of the anonymity set, and $p_{i}$ is the probability that an adversary can identify a node as a sender. If $p_{i}$ obeys a uniform distribution, that is,

(8) $$p_{i}=\frac{1}{N},$$

then we can obtain:

(9) $$H_{M}=\log_{2}N.$$

Subsequently, the adversary may exclude some improbable nodes through traffic analysis, timing attacks, and other attack methods. Then, the size of the new anonymity set is *S*.

At this time, $H_{S}=\log_{2}S$. The anonymity degree is:

(10) $$D_{1}=1-\frac{H_{M}-H_{S}}{H_{M}}=\frac{H_{S}}{H_{M}}=\frac{\log_{2}S}{\log_{2}N}.$$

Suppose the adversary fails to exclude any node, i.e., N=S. Then, the degree of anonymity is 1, indicating that the anonymous communication system has the most significant anonymity. On the other hand, suppose the adversary can determine the sender’s identity, i.e., S=1. Then, we can obtain that the degree of anonymity is 0, indicating that the anonymous communication system is compromised.

**In mTor and Conflux:** For multipath anonymous communication systems such as Conflux and mTor, we let *N* be the size of the anonymity set. Then, the maximum entropy of *N* users is:

(11) $$H_{M}=-\sum_{i=1}^{N}p_{i}\cdot \log_{2}p_{i}.$$

For Conflux or mTor with *m* paths, it contains *m* entry nodes and 1 exit node. Then, the probability that the adversary determines that the user is the sender is:

(12) $$p_{i}=\frac{m}{N}.$$

Then, the maximum entropy is:

(13) $$H_{M}=-\sum_{i=1}^{N}p_{i}\cdot \log_{2}p_{i}=m\cdot \log_{2}\frac{N}{m}.$$

The adversary can reduce the anonymity set by excluding some unlikely nodes through traffic analysis, timing attacks, and other attacks. The size of the new anonymity set is *S*. At this time,

(14) $$H_{S}=m\log_{2}\frac{S}{m}.$$

We can obtain the anonymity degree:

(15) $$D_{2}=\frac{H_{S}}{H_{M}}=\frac{\log_{2}S-\log_{2}m}{\log_{2}N-\log_{2}m}$$

**Figure 6:**
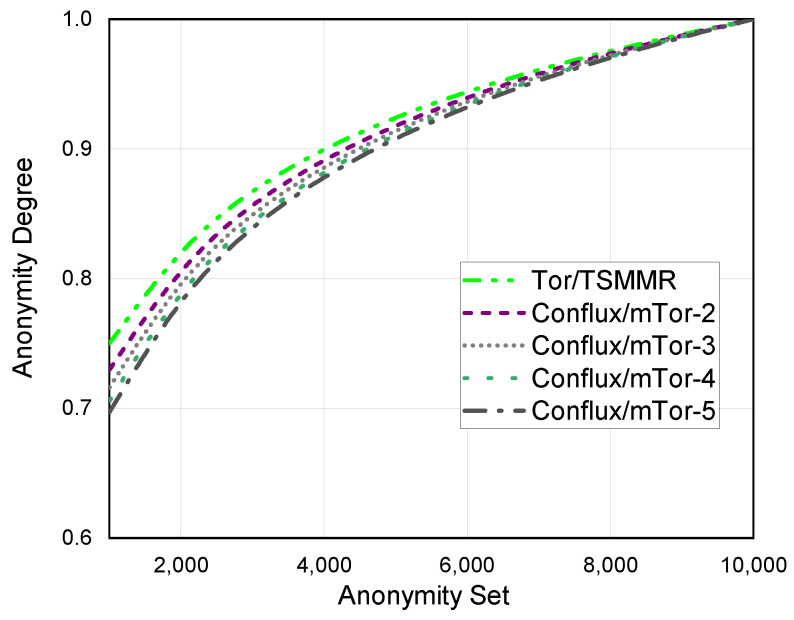
Anonymity degree of different anonymous communication networks.

We assume that there are 10,000 nodes in each anonymous communication network, and the maximum anonymity set is 10,000. As Figure 6 shows, we compare the anonymity degree of different anonymous communication networks when the anonymity set increases. When the multipath number m>1, it is evident that Tor and TSMMR have a more considerable anonymity degree than Conflux and mTor.

## 6. Conclusions

This paper aims to reduce the potential for congestion of anonymous communication networks. We use multiple middle relays to split the traffic and transmit it in parallel through multiple paths. Furthermore, we use low-bandwidth nodes as middle relays to transmit traffic. We introduce TSMMR’s design and compare its performance with other anonymous communication networks such as Tor, mTor, and Conflux. For the entry nodes, middle relays, and exit nodes on the anonymous communication path, we use different node selection strategies to select them respectively. Furthermore, we use different sending rates to simulate different types of users. The results show that the percentage of nodes with lower bandwidth utilization is larger in TSMMR than in other anonymous communication networks. Conversely, the portion of nodes with higher bandwidth utilization is smaller in TSMMR than in other anonymous communication networks. The results are the same when the multipath number is varied, indicating that TSMMR can provide better load balancing than other anonymous communication networks. In addition, we also compare the probability of anonymous communication paths being compromised (the adversary controls the entry and exit nodes at the same time). Assuming that the adversary cannot control all nodes, TSMMR and Tor have the same compromise probability and are better than Conflux and mTor. Finally, we evaluated the anonymity of different anonymous communication networks more generally. We calculate the anonymity degree of these anonymous communication networks. The results show that TSMMR can provide the same anonymity degree as Tor. Moreover, when the set of users is constant, the smaller the anonymity set is, and the better anonymity TSMMR an provide than other multipath anonymous communication networks. 

## Figures and Tables

**Table 1:** The percentage of nodes in different anonymous communication networks in different bandwidth utilization ranges when the sending rate is 50 kB/s.

Bandwidth Utilization	Tor	TSMMR-3	TSMMR-5	mTor-3	mTor-5	Conflux-3	Conflux-5
0∼30%	99.7%	100%	100%	84.9%	85.5%	99.8%	99.8%
30%∼80%	0.2%	0	0	0.5%	0.5%	0.1%	0.1%
80%∼100%	0.1%	0	0	14.6%	14%	0.1%	0.1%

**Table 2:** The percentage of nodes in different anonymous communication networks in different bandwidth utilization ranges when the sending rate is 100 kB/s.

Bandwidth Utilization	Tor	TSMMR-3	TSMMR-5	mTor-3	mTor-5	Conflux-3	Conflux-5
0∼30%	99.7%	100%	100%	84.6%	84.6%	99.6%	99.6%
30%∼80%	0.2%	0	0	0.3%	0.4%	0.2%	0.2%
80%∼100%	0.1%	0	0	15.1%	15%	0.2%	0.2%

**Table 3:** The percentage of nodes in different anonymous communication networks in different bandwidth utilization ranges when the sending rate is 500 kB/s.

Bandwidth Utilization	Tor	TSMMR-3	TSMMR-5	mTor-3	mTor-5	Conflux-3	Conflux-5
0∼30%	72.7%	84%	85.7%	74.9%	76.2%	82.4%	83.7%
30%∼80%	24.1%	15.9%	14.3%	8.6%	7.2%	16.4%	14.9%
80%∼100%	3.2%	0.1%	0	16.5%	16.6%	1.2%	1.4%

**Table 4:** The percentage of nodes in different anonymous communication networks in different bandwidth utilization ranges when the sending rate is 1000 kB/s.

Bandwidth Utilization	Tor	TSMMR-3	TSMMR-5	mTor-3	mTor-5	Conflux-3	Conflux-5
0∼30%	52%	64.5%	64.9%	49.3%	49.8%	61.4%	62.9%
30%∼80%	33.7%	30.7%	30.6%	30.7%	30.5%	32.5%	32%
80%∼100%	14.3%	4.8%	4.5%	20%	19.7%	6.1%	5.1%

## Data Availability

Not applicable.
